# High-fidelity synthesis of microhole templates with low-surface-energy-enabled self-releasing photolithography

**DOI:** 10.1039/d4ra00660g

**Published:** 2024-04-16

**Authors:** Peipei Jia, Shaolin Zhou, Xiaobing Cai, Qiuquan Guo, Haoran Niu, Wenping Ning, Yong Sun, Dongxing Zhang

**Affiliations:** a Shenzhen Institute for Advanced Study, University of Electronic Science and Technology of China Shenzhen Guangdong 518100 China yong_sun@uestc.edu.cn zhangdongxing@uestc.edu.cn; b School of Electronics and Information Engineering, South China University of Technology Guangzhou Guangdong China; c School of Aerospace Engineering, Xi'an Jiaotong University Xi'an Shanxi China

## Abstract

Material patterning through templates has provided an efficient way to meet the critical requirement for surface function in various fields. Here, we develop a self-releasing photolithographic process to make large-area freestanding templates with precise patterns. The low surface energy of substrates by hydrophobic treatment with proper silane modification ensures the template self-releasing. This method eliminates the need of mechanical separation or any sacrificial layers. Major steps including UV exposure and baking are optimized to realize high-quality structures and the final release of templates. The negative photoresists of SU-8 and polyimide are chosen to confirm the feasibility of this process. Wafer-scale freestanding templates with uniform microhole arrays are obtained with high structural fidelity, smooth surfaces and excellent flexibility. The hole size ranges from several to several tens of micrometers with an extremely low variation (<1%). These advantages could promote the application of precisely structured templates for surface patterning in material and surface science.

## Abstract 2

A self-releasing photolithographic technique is developed to synthesize freestanding polymeric templates with high structural fidelity, smooth surfaces and excellent flexibility.

## Graphical abstract

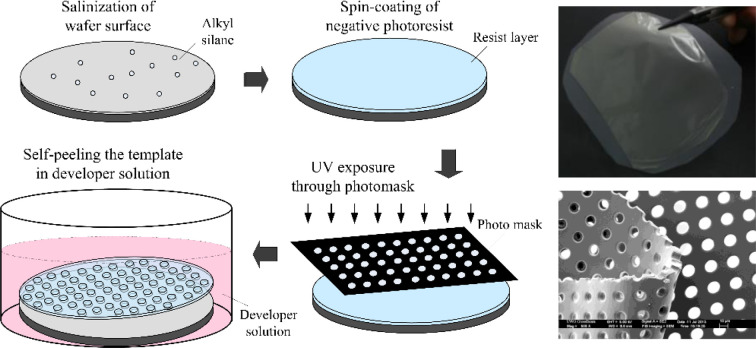

## Introduction

As a versatile surface engineering technique, patterning with structured templates is of fundamental importance in the fields of material science, biotechnology and nanotechnology. Specifically, freestanding templates based on thin membranes with well-defined micro/nanoholes are highly demanded as shadow masks for material synthesis in the biological assay of cells, DNA and proteins,^1–3^ chemical assisted emulsification,^4,5^ and nanomaterial patterning on arbitrary substrates otherwise incompatible with conventional techniques.^6,7^

Diverse materials have been patterned onto target surfaces by synthesis and deposition through templates. For instance, a dry lift-off procedure with a elastomeric template was demonstrated to pattern metals, organometallic molecules, biological macromolecules, and sol–gels.^8^ With a soft-lithographic template/stencil sealed on the substrate, topologically designable microstructures were realized with chemical vapour deposition (CVD) and reactive coating deposition.^9,10^ Metallic nanohole arrays were produced by a template-transfer procedure with nanostructured Si templates for plasmonic sensing.^11,12^ SU8 templates with micro-posts were utilized for nanoparticle tracking under optical microscope at biomolecular and cellular levels.^13^ Beyond the use in fabrication, structured templates are also incorporated into microfluidic system to act as key components such as separators,^14,15^ multiplexors,^16^ and controllable interconnects for different channels/levels of microfluidic arrays.^17^

Advance of fabrication technology has continually driven the evolution of template modality. Typical membrane templates made by phase separation usually suffer from irregular porous structures in random distribution.^18^ In contrast, the track-etched^19,20^ and anodic aluminium oxide (AAO)^21,22^ membranes feature the narrower hole-size distribution and lower tortuosity. However, the use of nuclear fission or corrosive chemicals induces laborious procedures and undesirable contaminations. As a result, the more advanced routes are employed to fabricate templates with ideal hole geometry, including the micromolding,^23–27^ interference lithography,^28–30^ soft lithography^6–8,31^ and the chemical assembly techniques such as particle-assisted wetting^32,33^ and emulsion templating.^34,35^ However, one particular difficulty lies in the separation of templates from the master structures due to the resistance force from microholes, resulting in potential structural failures.^36^ Besides, consistent fabrication of large-area templates is quite challenging owing to the complication between the applied pressure and the viscosity of the pre-polymer.^37^

In this paper, we present a self-releasing photolithographic technique for large-area fabrication of structured templates. Before patterning templates, the surface modification with specific silanes is used to turns the substrate to be hydrophobic. This low energy surface enables the structured template to self-release during the development. This procedure is naturally combined into the conventional photolithography processes, eliminating the need of mechanical separation or any sacrificial layers. Negative resist SU-8 and the photo-definable polyimide are selected for microhole template fabrication. With the full advantage of photolithography, this method paves a simple way of high-fidelity fabrication of templates for their application in micro/nano fabrication, chemistry and biomedical science.

## Experimental

Two types of the negative photoresists used in our experiment are SU8-3010 (Microchem, USA) and the photo-definable polyimide precursor HD 4100 (HD Microsystems™, USA). Notably, SU-8 is widely used in nanofabrication, microfluidics and soft lithography due to its chemical and mechanical stability; the templates with nontoxic and well biocompatible polyimide could benefit biological or biomedical applications.

**Fig. 1:**
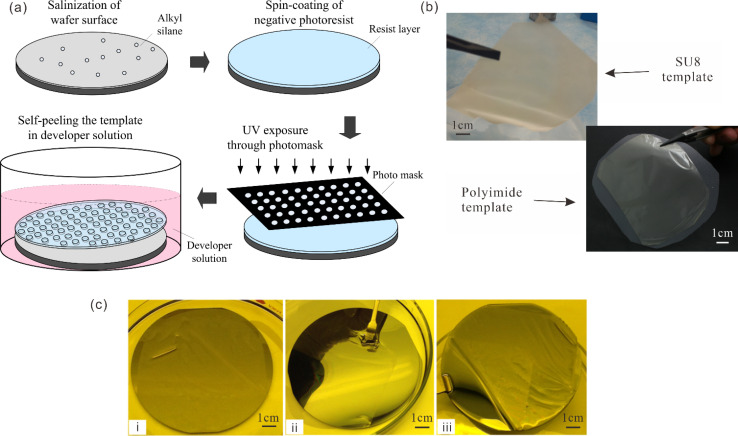
(a) Schematic of template fabrication by self-releasing photolithography. (b) Wafer-scale freestanding microhole templates made of SU8 (top) and polyimide (bottom). (c) Structured template self-releasing process: (i) self-detaching during development, (ii) freestanding on wafer surface in the developer, and (iii) template supported by wafer surface after rinsing.

A brief illustration of template fabrication process is shown in Fig. 1a. A substrate of silicon wafer is first cleaned by heated Nano-Strip (Cyantek, CA, USA) at 85 °C to remove any contaminations including organic and inorganic particles and rinsed by DI water. After the dehydration on hotplate at 200 °C for 5 minutes, the wafer surface is silanized using alkyl silanes under vacuum for 1 h to decrease the surface energy to enable self-peeling of patterned template during resist development. As an example of the typical procedure, SU-8 3010 is spin-coated at 500 rpm for 5 seconds followed by 1000 rpm for 30–35 seconds onto the silanized wafer using a spin-coater (Solitec 5110 Spinner) for the final template thickness around 15 μm. After soft baking at 95 °C for 5 minutes, the resist-coated wafer is exposed by the 365 nm UV light for an energy dose of 180 mJ cm^−2^ through a pre-designed photomask in a contact lithographic system (Karl SyssMA6 Mask Aligner, UV intensity of 12 mW cm^−2^). The post-baking is performed at 65 °C for 2.5 minutes followed by 95 °C for another 2.5 minutes directly after exposure. The resist layer is then developed in the SU-8 developer solution in a Petri dish. The crosslinked template gradually self-peels from the wafer surface without any other intervention. The synthesized freestanding template is cleaned again with fresh SU-8 developer and isopropyl alcohol, and finally stored in a ventilated cabinet for slow dehydration to prevent it from wrinkling. Fig. 1b demonstrates the freestanding and flexible templates of SU8 and the polyimide in wafer scale.

## Technical discussion

The same fabrication procedures with different parameters are applicable for SU8 and polyimide respectively. However, two major issues exist in the fabrication: (1) the low adhesion due to silanization could prevent uniformly spin-coating resist layers; (2) overexposure may lead to saturated structures. Thus, the silanization and exposure dose need to be adjusted according to different adhesion and optical sensitivity of two resists, respectively.

**Fig. 2:**
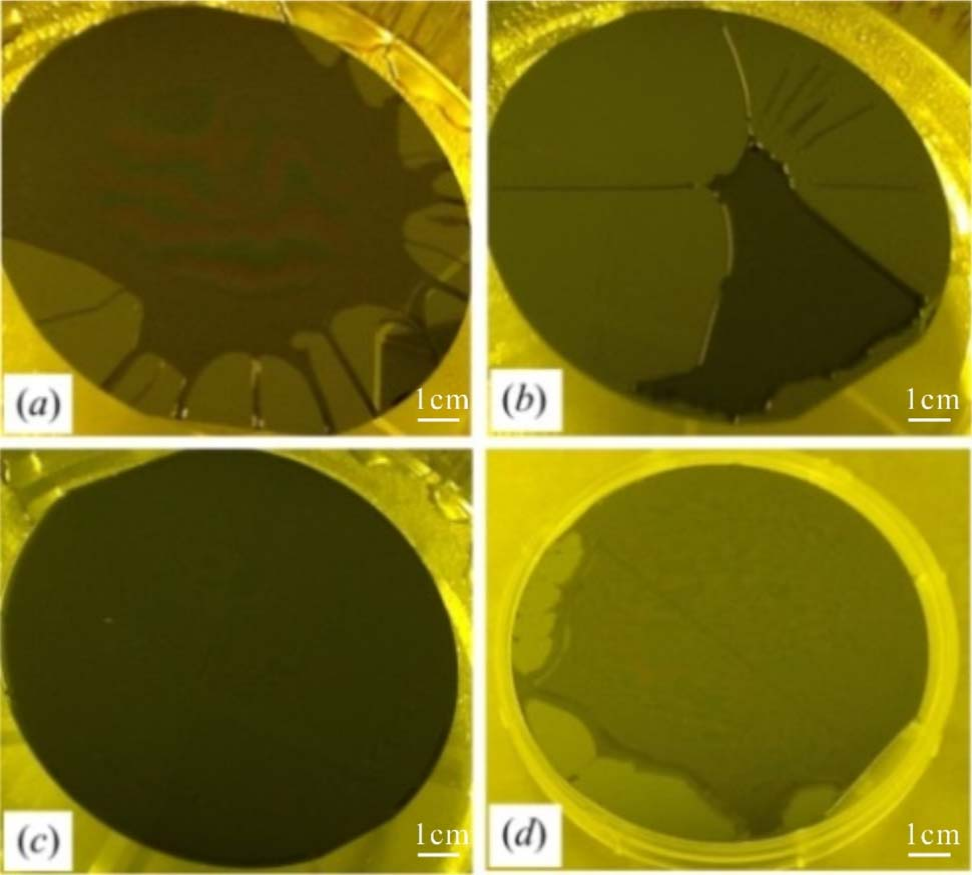
Silanization test by spin-coating photoresists. (a) The SU8 3010 and (b) polyimide HD 4100 spin-coated onto wafer surfaces silanized by FOTS. (c) Polyimide HD 4100 uniformly spin-coated onto wafer surface silanized by *n*-OTS. (d) Polyimide HD4100 in (c) baked to 90 °C by an uncontrolled increase of temperature.

First of all, the proper silanization of substrate surfaces is essential for large-area photolithographic fabrication of freestanding templates. The substrate needs to be treated to certain degree of hydrophobicity according to the used photoresist, whereas inappropriate treatment may cause non-uniform resist spin-coating or failure in the final self-releasing of patterned templates. Trichloro-(1*H*,1*H*,2*H*,2*H*-perfluorooctyl)silane (FOTS) and *n*-octadecyltrichlorosilane (*n*-OTS) are compared in our silanization test by spin-coating the selected photoresists on substrates. Fig. 2a and b show the photoresist dewetting on the wafer surfaces silanized by FOTS, indicating the excessively low surface energy. The size comparison of dewetting areas implies that SU-8 (Fig. 2a) is more adhesive to the substrate with the same degree of hydrophobicity than the polyimide (Fig. 2b). In contrast, the wafer surface treated with *n*-OTS show less hydrophobicity, thereby facilitating the resist spin-coating and archiving the complete surface cover, as shown in Fig. 2c. Notice that temperature should be slowly increased in soft baking, otherwise the dramatic temperature rise can crack the resist layer near the edge, as shown in Fig. 2d. Moreover, the baking temperature should been set according to the recommendation to guarantee the thermal stability of the resist.^38^

**Fig. 3:**
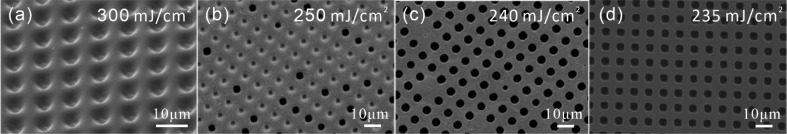
UV dose control for high-fidelity fabrication of polyimide templates with microholes: exposure energy decreasing for scanning electron microscopy (SEM) images from (a) to (d).

To obtain high-fidelity perforated structures in templates, the UV dose control is another critical measure. On the one hand, the complete crosslink throughout the negative photoresist layer requires a minimum energy dose, under which the catalyzer can reach the bottom region, enabling crosslink of the entire photoresist layer. On the other hand, overexposure may further cure the resist of the undesired area, leading to template structure failure. For instance, the holes that are supposed to perforate the template become closed, as shown in Fig. 3a. Since the polyimide is more susceptible to UV energy variation, the exposure test is carried out to obtain the optimum dose for templates with the hole feature as small as 5 μm (Fig. 3). In contrast, SU8 templates has a larger window of exposure dose as SU8 is relatively less sensitive to overexposure. However, SU8 template tends to be more adhesive to substrate surfaces. Slight overexposure (*e.g.* 200 mJ cm^−2^ for the thickness of 15 μm) can strengthen its adhesion to substrates, thereby impeding the final delamination of the structured template.

Others factors such as baking times are also controlled according to the template thickness and photoresists. In traditional photolithography, as the cured photoresist remains on substrates to form micro/nanostructures, the longer baking time is usually helpful to enhance the strength of final structures. In our case, for the sake of releasing the crosslinked template from substrate, the post-baking is optimized to such an extent that guarantees both crosslinking and self-peeling of the template. Especially, post-baking time needs to be taken good control for SU8, because over-baking can significantly strength its adhesion to the substrate.

## Characterization of templates

**Fig. 4:**
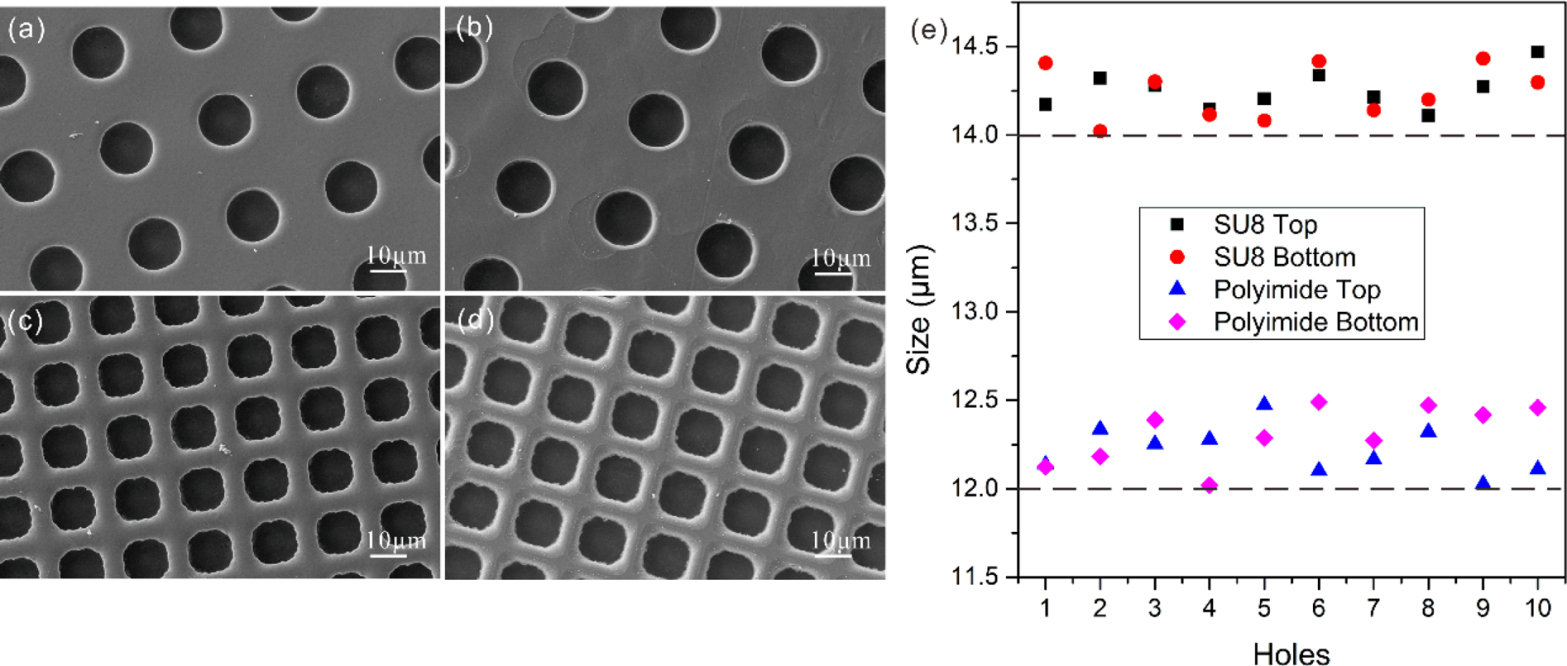
SEM images of top and bottom of SU8 (a and b) and polyimide (c and d) templates. The diameter of round SU8 hole and the edge of square polyimide holes are 14 μm and 12 μm, respectively. (e) Size measurement of each 10 holes from top and bottom of SU8 and polyimide templates respectively.

For uniform patterning of materials, the high-quality template needs to possess well-defined hole size, shape and distribution. This requirement is accurately realized in our templates with the optimized process. The patterned hole sizes range from several micrometers to several tens of micrometers. Correspondingly, the porosity of templates can reach ∼10^5^ holes per cm^2^. Fig. 4 show the SEM images of freestanding templates made of SU8 and the polyimide. For the high-resolution SU8 template, 14 μm diameter of the round holes has only a standard deviation of ∼100 nm based on a random measurement of 10 holes (Fig. 4e). The coefficient of variation is calculated to be less than 1%. In contrast, the micromoles formed by track etch and aperture array lithography show a 15% and 7% coefficient of variation, respectively. In addition, the hole sizes on the top and bottom of one template remain nearly unchanged. The only difference is that the bottom surface has a few residue particles and slight flaws, probably due to the incomplete crosslink of photoresist (Fig. 4b and d). Note, as the polyimide is sensitive to UV energy variation, a bit lower dose is applied to ensure all the microholes open. However, this underexposure also results in the rough edges of the square holes in the polyimide template (Fig. 4c and d).

**Fig. 5:**
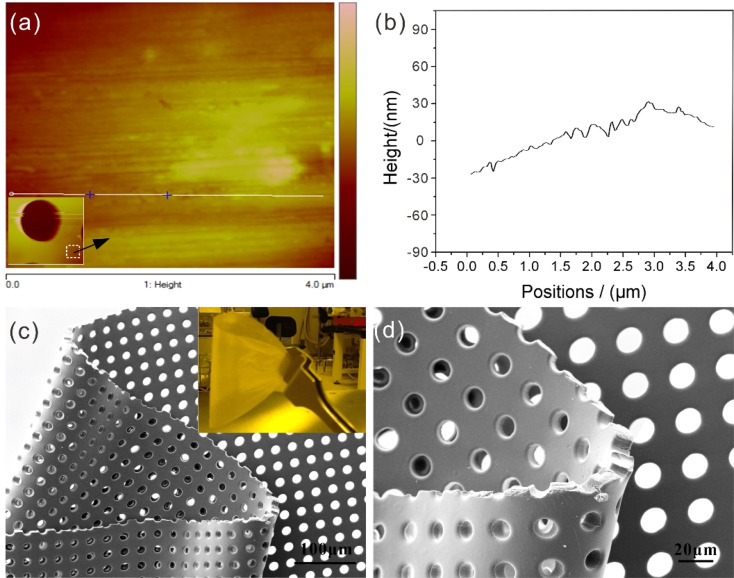
Roughness and bending tests of template with high-fidelity microholes. (a) Topography analysis of AFM measurements of the bottom surfaces (4 × 4 μm^2^) of the SU8 template. The white square in the inset indicates the scan areas of (a). The height profiles (b) shows the roughness within ±30 nm along the line in (a). (c) SEM image of threefold bended SU8 template ((inset): bending test) and enlarged image (d) with intact microholes.

Further, the template surface is also required to be smooth and flexible to ensure a close attachment to the target substrate for material deposition. The surface profile of our templates is quantified using atomic force microscopy (AFM). The roughness of the SU8 bottom surface (Fig. 5a) are within ±30 nm (Fig. 5b). The root mean square of roughness over a 4 × 4 μm^2^ are 13.3 nm for the bottom surfaces. Our thin templates show remarkable flexibility in the bending and stretching test as well. The insert in Fig. 5c shows the template upon bending, in which it remains intact after 50 cycles. Usually, such a thin template without a support could be easily distorted or even damaged due to internal tension. In contrast, the microholes in our template maintain their original shape and arrangement even under threefold bending (Fig. 5c and d). By comparison, the polyimide template turns out to be more extendable with higher mechanical strength, whereas the SU8 template is more flexible.

## Material patterning with templates

**Fig. 6:**
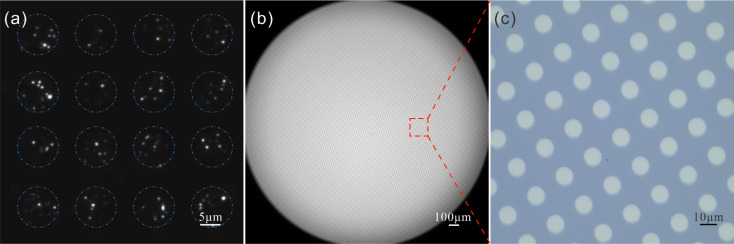
Patterning polystyrene nanoparticles and metal disks with microhole templates. (a) Fluorescent image of nanoparticle distribution in an array according to the used SU8 template. (b) Microscopy image of large-area platinum micro-disk array and enlarged image (c) with precise arrangement and dimensions.

We demonstrate the versatility of our templates by material patterning in both liquid and gas phases. The distribution of nanoparticles can be confined by their solution deposition through microhole templates. An SU8 template and a glass slide are first treated with oxygen plasma immediately before their contact, enabling a close bond between them. 100 nm fluorescent polystyrene particles in anhydrous ethanol are dropped on the template to generate a pattern enriched with nanoparticles on the glass surface after ethanol evaporation. As shown in Fig. 6a, nanoparticles are allocated in an array according to the template without leakage. Besides, our templates are also applicable to bulk material patterning through physical vapor deposition. For example, platinum can be sputtered onto the substrate *via* the SU8 template to produce a micro-disk array (Fig. 6b and c). The uniform array can be generated with precise arrangement and dimensions as those of microholes in large area.

## Conclusions

In conclusion, we develop a self-releasing photolithographic technique to synthesize freestanding polymeric templates with precise microholes. Our method eliminates sacrificial layers used in previous techniques to enable self-detachment of template from substrates. Instead, the self-releasing relies on the salinization of substrate surfaces to proper hydrophobicity, ensuring both uniform spin-coating of resists and detachment of templates. Moreover, this process is fully compatible with the conventional photolithography, taking all its advantages to engineer specific microstructures, *e.g.* an extremely low variation. Major steps including UV exposure and baking are also precisely controlled to realize high-quality patterning. As a result, wafer-scale templates with uniform microhole arrays are obtained with high structural fidelity, smooth surfaces and excellent flexibility. Our templates are capable of patterning nanoparticles and bulk material by solution deposition and physical vapor deposition respectively. These advantages could extend the application of precisely structured templates for surface patterning of various materials in more scientific and engineering areas.

## Author contributions

Peipei Jia: manuscript writing, data curation, methodology and funding. Shaolin Zhou: experimental, manuscript writing, data curation and methodology. Xiaobing Cai and Qiuquan Guo: data curation and methodology. Yong Sun and Dongxing Zhang: funding and supervision.

## Conflicts of interest

There are no conflicts to declare.

## Supplementary Material
